# Effects of forage feeding level on ruminal pH and metabolic adaptation of the rumen epithelium in pre-weaned Jersey calves

**DOI:** 10.1016/j.vas.2024.100384

**Published:** 2024-07-26

**Authors:** Madeline N. Bennett, Dana E. McCurdy, Anne H. Laarman

**Affiliations:** a Department of Animal, Veterinary and Food Science, University of Idaho, Moscow, 83844, United States; b Department of Agricultural, Food and Nutritional Science, University of Alberta, Edmonton, AB, T6G 2P5, Canada

**Keywords:** Rumen pH, Forage, Dairy calf, Weaning

## Abstract

The objective of this study was to determine the effect of limiting forage provision in pre-weaned calves on ruminal pH and short chain fatty acid (SCFA) transport capacity during the pre-weaning period. Twelve Jersey bull calves (age = 1.9 ± 0.8 d) were housed individually on sand. All calves were fed milk replacer at 1,200 g/d and texturized grain-based starter ad libitum from birth. Calves were randomly assigned one of two treatments: ad libitum forage (**ALF**) or limited forage provision, where forage was limited to 90 g/d as-fed (**LFP**). Individual feed intake was recorded daily, calf weights, and jugular blood samples were collected weekly. Once calves consumed 680 g/d of calf starter, ruminal pH was measured for seven days after which calves were humanely killed and rumen fluid sampled. During the pre-weaning period, starter intake, feed efficiency, plasma glucose and β-hydroxybutyrate (BHB) concentration, SCFA concentration, average daily gain, and body weight were not different between treatments. Forage intake for ALF calves was greater than LFP beginning at wk 9 (255 ± 34 vs. 71 ± 40 g/d, respectively). Compared to ALF, LFP decreased mean ruminal pH (6.38 ± 0.16 vs. 5.98 ± 0.23) and duration of time where rumen pH was below 5.8 (796 ± 145 vs. 261 ± 133 min/d). Epithelial markers of SCFA transport and cell homeostasis (MCT1, NBC1, NHE3) were not affected by treatment. In conclusion, incidence of sub-acute ruminal acidosis in limited forage-fed calves did not have the same effects on intake and nutrient transporters seen in adult cows.

## 1 Introduction

Calves are born with a nonfunctional rumen and must transition major energy sources and develop the rumen before the removal of milk during weaning. The principal route of rumen development is through the intake and fermentation of solid feed, leading to production of short chain fatty acids (SCFA) that promote development of the rumen papillae (Gorka et al., 2018). Ensuring calf starter intake of at least 680 g/d (NRC, 2001) or 1.0 kg/d (Stamey et al., 2012) prior to weaning is recommended as a proxy of sufficient rumen development to allow for successful weaning (Stamey et al., 2012). Such high intakes of rapidly fermentable calf starter risks depressing rumen pH if forage in the diet is absent.

In calves, rumen pH ranges between 5.5–5.9 for 7–18 h/day, irrespective of age and diet (Laarman et al., 2012; van Niekerk et al., 2021; Wood et al., 2015). In cows, pH of 5.5 – 5.9, known as subacute ruminal acidosis, is associated with reduced feed intake and milk production and numerous adverse health events (Plaizier et al., 2018). In calves, the impact of low rumen pH is unclear as it is unrelated to starter intake, papillae size, corneum thickness, or acute phase proteins (Kristensen et al., 2007; van Niekerk et al., 2021). Additionally, low rumen pH can increase average daily gain in calves (Laarman et al., 2012; McCurdy et al., 2019), suggesting rumen pH has differential impacts in calves and cows.

In calves reared in North American production systems, where grain-based starters and dry forages are the norm, rumen pH is positively correlated to dry forage intake before and after weaning (Laarman & Oba, 2011; Laarman et al., 2012). Forage intake, whether bedding or provided forage, appears to be negligible until 5 weeks of age (Suarez-Mena et al., 2016), even when calf starter is introduced. Several studies concluded that 5 to 10 % forage inclusion in pre-weaning diets improves dry matter intake, body weight gain, and reduces non-nutritive behaviors (Aragona et al., 2021; Horvath et al., 2020; Jahani-Moghadam et al., 2015). Similarly, in 7-wk old Holstein calves with ad libitum access to timothy hay or mixed legume hay, occurrence of subacute ruminal acidosis was eliminated when daily forage intake exceeded 80 g/d DM (Laarman & Oba, 2011), corroborating 5–10 % forage intake as being essential. Whether 10 % forage intake increases rumen pH for all calves, however, is unclear.

To aid in the understanding of forage provision to calves and rumen pH on cellular development, the objectives of this study were to: 1) determine the effect of limiting forage provision in pre-weaned calves on starter intake and ruminal pH; and 2) determine the impact of rumen pH on SCFA transport capacity and pH_i_ homeostasis capacity during the pre-weaning period. We hypothesized that limiting forage intake will not affect starter intake, rumen pH or SCFA transport in pre-weaned calves.

## 2 Materials and methods

### 2.1 Animals, management, and treatments

All animal work was approved by the University of Idaho Institutional Animal Care and Use Committee (AUP 2016-16). Animal work occurred between December 2016 and March 2017.

Jersey bull calves (*n* = 21; age = 0 – 5 d) from a commercial farm near Twin Falls, Idaho, were fed 4 L of colostrum within 3 h of birth and were transported 670 km to the University of Idaho's Palouse Research, Extension, and Education Center, where they were used for a separate study on transport stress (Chibisa et al., 2018). Calves were castrated prior to arrival as per commercial farm protocol but were not disbudded or weaned over the course of this study. Upon arrival, all calves were treated with ampicillin (0.90 mg/kg of body weight) for 3 days at the recommendation of the attending veterinarian.

All calves were individually housed in a pole barn in hutches (Calf Nursery, Polydome Inc., Litchfield, MN) with sand for bedding; calves were provided ad libitum access to water and commercial, grain-based texturized starter (89.1 % DM, and 21.1 % CP, 36.5 % starch, 14.6 % NDF, 7.6 % ADF on DM basis; AMPLI-CALF Starter 20, Land O'Lakes Purina Animal Nutrition, Tulare, CA) via hutch bucket; water and starter were refreshed daily at 0700 h and water was replenished again at 1800 h. Calves were bottle-fed 900 g/d as-fed of milk replacer (28 % CP/18 % fat, DM basis; Calva Advantage, Calva Products LLC, Acampo, CA; Table 1) twice daily at 0700 and 1800 h in equal allotments with 15 % solids. If a milk replacer refusal exceeded 300 g (2 L), refusals were fed through esophageal tubing. Calves with reduced feed intake or signs of dehydrations or scours were offered electrolytes via tube feeding (RE-SORB, Zoetis Services LLC., Parsippany, NJ) twice daily in addition to allotted milk replacer. If milk replacer intake was complete and calves appeared hungry, milk allotment was increased 75 g (500 mL) until daily intakes of milk replacer reached 1200 g/d. When weekly average ambient temperature was less than 0 °C, an energy supplement was added to the milk replacer at 20 g/d as-fed (7 % CP/60 % fat, DM basis; K-Cal Energy Supplement, Calf Solutions Products, Chilton, WI).

**Table 1:** Feed intake of pre-weaned Jersey calves^1^ provided with long-stem alfalfa hay either ad libitum (ALF) or limited forage provision at 90 g/d, as-fed (LFP).

Measurement	ALF^2^	LFP^2^	*P*-value
Overall			
Average daily gain, kg/d	0.91 ± 0.03	0.89 ± 0.04	0.61
Gain:Feed	0.62 ± 0.04	0.64 ± 0.05	0.76
			
From weaning readiness^3^			
Age, d	82.9 ± 6.1	96.3 ± 8.1	0.08
Milk replacer intake, g/d	1200	1200	N/A
Starter DMI, g/d	617 ± 90	763 ± 106	0.33
Hay DMI, g/d	375 ± 62	62 ± 73	< 0.01
DMI,%BW	0.91 ± 0.09	0.68 ± 0.11	0.05
			
Hay intake,%Total DMI	37.0 ± 5.8	8.5 ± 6.9	0.01
Forage NDF intake, g/d	167 ± 28	28 ± 33	< 0.01
NDF intake, g/d	257 ± 25	139 ± 29	0.01
Starch intake, g/d	232 ± 32	279 ± 38	0.37

1 All calves were individually housed in hutches inside a pole barn, using sand as bedding. All calves were fed 1200 g/d of milk replacer and had ad libitum access to water and calf starter.

2 n = 6.

3 Weaning readiness was defined as a calf starter consumption of at least 680 g/d for 3 consecutive days.

At 14 d of age, calves were assigned one of two forage provision treatments: control calves were provided with ad libitum access to single-sourced long-stem alfalfa hay (**ALF**; 79.8 % DM, and 17.7 % CP, 44.5 % NDF, 37.6 % ADF, and 41.6 % TDN on DM basis), whereas limit-fed calves were provided 90 g/d as-fed of long-stem alfalfa hay (**LFP**); hay was provided in 20 L bucket on the ground, fastened to calf hutch, next to the starter and water buckets. Feed intakes were recorded daily; summed weekly values were used in statistical analyzes. Weekly, calves were weighed, and a jugular blood sample was collected within 3 h of feeding for later blood glucose and BHB analysis.

Once a calf was ready to wean, as indicated by consumption of 680 g/d of starter as-fed for three consecutive days (NRC, 2001), a calf was dosed with a stand-alone, submersible ruminal pH logger for small ruminants (T-9, Dascor Inc., Escondido, CA) and rumen pH was recorded continuously for 7 d without a reduction in milk provision. The 7-d collection period allowed for representative data calculation that incorporates day-to-day variation without risking pH sensor failure. At the conclusion of day seven, calves were euthanized by captive bolt stunning and exsanguination at 0900 h, 2 h after morning feeding; a blood sample was collected at harvest from mixed jugular and arterial flow for later analysis of glucose and BHB. Rumen pH logger was retrieved and calibrated post-measurement to correct for drift; rumen contents were sampled from the ventral sac and squeezed through four layers of cheese cloth and snap-frozen in liquid nitrogen for SCFA analysis as described previously (Laarman et al., 2012).

### 2.2 Sample analysis

Fresh feed samples were collected from five different locations each time a new bag of milk replacer, bag of calf starter, or bale of long-stem alfalfa hay was used. All samples were ground to 1 mm, composited, and were analyzed by a commercial lab (Cumberland Valley Analytical Services, Waynesboro, PA). Blood was centrifuged at 3000 × *g* for 20 min. at 4 °C, and plasma was aliquoted and frozen at -20 °C for metabolite analysis. Rumen pH data (7 consecutive d) from each calf were downloaded, and minimum pH, mean pH, maximum pH, duration of subacute ruminal acidosis (pH < 5.8) and area under the curve of subacute ruminal acidosis were calculated for each of the 7 d, then the 7 values were averaged to account for day-to-day variation. Commercial enzymatic kits were used for glucose (Wako Diagnostics, Mountain View, California), and BHB (Wako Diagnostics Inc., Mountain View, CA) analysis on a SpectraMax i3x plate reader (Molecular Devices LLC., San Jose, CA). Rumen fluid collected at slaughter was processed for SCFA analysis as described previously Laarman et al., 2012), using an Agilent 6890 series gas chromatograph with a DB-FFAP column.

Rumen tissue samples were analyzed using immunofluorescence as previously described (Laarman et al., 2013). Briefly, all slides were blinded prior to analysis. Then samples were deparaffinized, placed in an antigen retrieval buffer (3 % sodium citrate solution) for 15 min at 95 °C, before being cooled to room temperature, rinsed with phosphate-buffered saline (PBS), then incubated with a 10 % goat serum and 0.3 % Triton-X100 blocking buffer for 30 min. Next, primary polyclonal antibodies (Novus Biologicals, Centennial, CO) were added to the slides for monocarboxylate transporter, isoform 1 (MCT1; 1:200 dilution), sodium bicarbonate cotransporter isoform 1 (NBC1; 1:200 dilution), and sodium/proton exchanger isoform 3 (NHE3; 1:100 dilution), each markers of rumen epithelial development (Hiltz et al., 2021; Laarman et al., 2012a; Yohe et al., 2019) and incubated at room temperature for 90 min. Slides were then rinsed with PBS, and a fluorescent secondary antibody (Invitrogen; 1:200 dilution) was added and incubated in the dark for 30 min, rinsed with PBS, and mounted with a DAPI nuclear stain (Prolong Gold Anti-fade, Cell Signaling Technologies, Danvers, MA). A negative control without primary antibody was used for each primary antibody. After staining was complete, slides were stored at -20 °C until they were analyzed.

Analysis of immunofluorescence was conducted using a confocal spinning disk microscope (Nikon TiE inverted microscope; Yokogawa XI Spinning Disk). The microscope settings were adjusted for each antibody to optimize visualization and kept the same within each antibody. For each slide, three different papillae, and three different areas on each papilla were imaged. Within each image, five cells were quantified and if abundance CV values were greater than 10 %, another set of five cells were quantified. Quantification of transporter abundance was done in ImageJ software by drawing around the perimeter of each cell (defined as the “relative cell circumference”) to receive the entire antibody signal within each cell perimeter (defined as “cell area”). A section of area adjacent to the papillae was used to calculate corrected whole cell signal. The formula used was (Gavet & Pines, 2010):

$$WCS=ID_{cell}-\left(A_{cell}\;\times \;M_{background}\right)$$

Where WCS = Whole Cell Signal; ID_cell_ = integrated cell density; A_cell_ = cell surface area; and M_background_ = mean background signal.

### 2.3 Statistical analysis

An outbreak of salmonellosis in wk 3 caused nine deaths and, for remaining calves (ALP *n* = 6; LFP *n* = 6), caused all intakes to drop for several weeks. Necropsies and fecal samples confirmed the presence of *Salmonella* spp. isolates in the intestines of four of nine dead calves and the feces of six surviving calves. With 12 animals remaining in the study (ALP *n* = 6; LFP *n* = 6), we retained sufficient power to detect 20 % differences between treatment with a 10 % coefficient of variation and 80 % power (Berndtson, 1991).

Data that were collected at multiple time points (blood metabolites and feed intake) were analyzed using repeated measures in PROC MIXED in SAS (SAS 9.4; SAS Inst. Inc., Cary, NC) according to the following model:

$$Y\;=\;\mu \;+\;t_{i}\;+\;w_{j}\;+\;t\;\times \;w_{\text{ij}}\;+\;\varepsilon_{\text{ijk}}$$

Where Y is the response, μ is the overall mean, t_i_ is the treatment effect, w_j_ is the time effect, t × w_ij_ is the interaction between time and treatment, and ε_ijk_ is the residual term. The variance-covariance structure of the repeated measures was modeled separately with an appropriate structure fitted using the lowest values of the fit statistics based on the Akaike Information Criterion.

Average daily gain was determined using PROC REG in SAS (SAS 9.4; SAS Inst. Inc., Cary, NC) by linearly regressing body weight against age over the duration of the study. For rumen pH, daily profiles were created for each of the 7 days. For each calf, the seven daily profiles were treated as technical replicates and averaged to incorporate day-to-day variation, yielding one biological replicate (Laarman & Oba, 2011).

Data that were collected or calculated once and yielded one biological replicate (average daily gain, age at weaning readiness, rumen pH, feed efficiency, SCFA profile, transporter abundance) were analyzed using the MIXED procedure of SAS (SAS 9.4; SAS Inst. Inc., Cary, NC) according to the following model.

$$Y\;=\;\mu \;+\;t_{i}\;+\;\varepsilon_{\text{ij}}$$

Where Y is the response, μ is the overall mean, t_i_ is the treatment effect, and ε_ij_ is the residual term. Significance was declared at *P* ≤ 0.05 for the mean ± standard error of measurement, and tendencies were declared at 0.05 < *P* ≤ 0.10.

R version 3.6.1 was used for all transporter statistical analysis (R Core Team, 2019). A Shapiro test was used to test the normality (*P* > 0.05) of sample distributions of MCT1 (*P* = 0.03 LFP, *P* = 0.66 ALF), NHE3 *(P =* 0.07 ALF, *P* = 0.02 LFP), and NBC1 *(P =* 0.12 ALF, *P* = 0.13 LFP), and a Fligner test was used to determine the homogeneity (*P* > 0.05) of variances *(P =* 0.86, 0.42, 0.33, respectively). A Kruskal-Wallace test was used to analyze MCT1 and NHE3 due to non-normal distributions of samples and a homogeneity of variances. An ANOVA test was used to analyze NBC1 due to a normal sample distribution and homogeneity of variances.

## 3 Results

### 3.1 Production performance

**Fig. 1:**
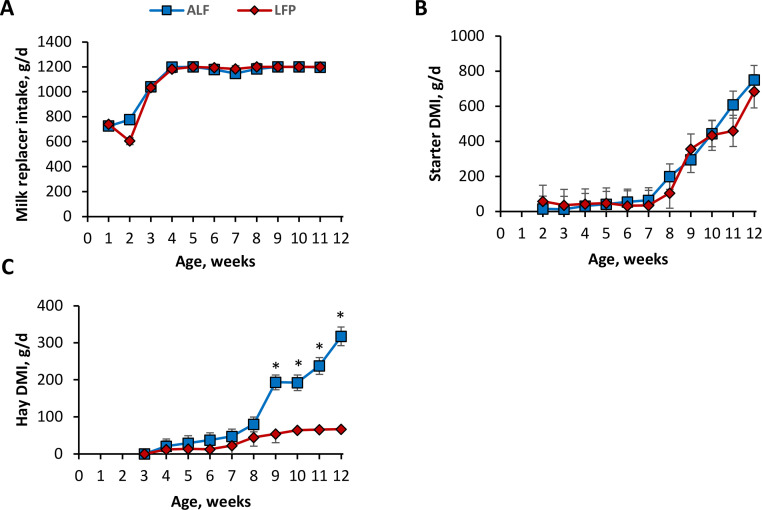
Milk replacer intake (A), starter DMI (B), and hay DMI (C) of pre-weaned Jersey calves provided with long-stem alfalfa hay either ad libitum (ALF) or limited forage provision at 90 g/d, as fed (LFP). All calves were individually housed in hutches inside a pole barn, using sand as bedding. All calves were fed 1200 g/d of milk replacer and had ad libitum access to water and calf starter. There was no difference in milk replacer intake (*P =* 0.43) or in starter intake (*P* = 0.82). Hay intake showed a treatment × time interaction (*P* < 0.01). *P < 0.05 between ALF and LFP.

Starter intake was similar between treatments for the duration of the study (*P* = 0.67; Fig. 1) and increased as calves aged (*P* < 0.01). During wk 2 to wk 8 of age, forage intake did not differ between treatments. Starting in wk 9, total forage intake in ALF was greater than LFP (193 ± 20 vs. 54 ± 24 g/d DMI, *P* < 0.01), as LFP calves were limited to 90 g/d as-fed (71.8 g/d DM). Calves in LFP reached the ceiling of 90 g/d as-fed in wk 10. Thereafter, ALF calves increased forage intake weekly until a maximum difference in forage intake in wk 12 between ALF and LFP (317 ± 25 vs. 67 g/d, *P* < 0.01).

**Fig. 2:**
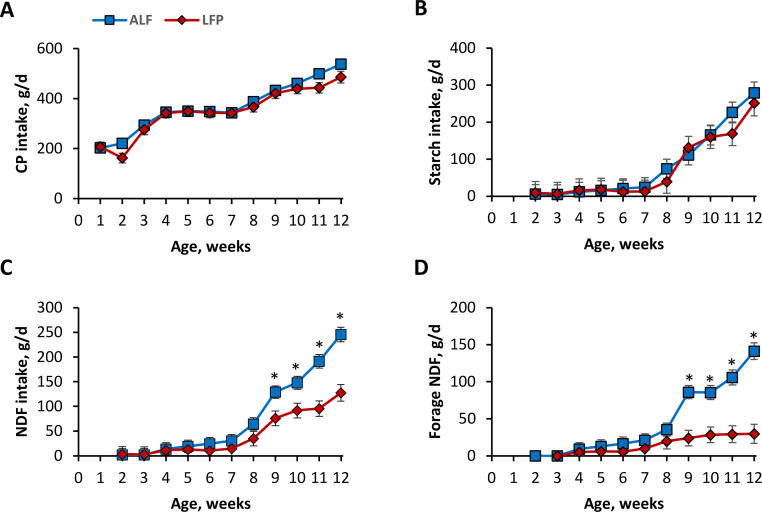
CP intake (A), starch intake (B), total NDF intake (C), and forage NDF intake (D) of pre-weaned Jersey calves provided with long-stem alfalfa hay either ad libitum (ALF) or limited forage provision at 90 g/d, as fed (LFP). All calves were individually housed in hutches inside a pole barn, using sand as bedding. All calves were fed 1200 g/d of milk replacer and had ad libitum access to water and calf starter. There was no difference in CP intake (*P =* 0.20) or in starch intake (*P* = 0.17). Total NDF intake and forage NDF intake showed a treatment × time interaction (*P* = 0.03 and *P* < 0.01, respectively). *P < 0.05 between ALF and LFP.

Between ALF and LFP, there was no difference in CP intake (*P* = 0.20) or in starch intake (*P* = 0.17; Fig. 2). Until wk 8, NDF intake was similar between ALF and LFP (30 ± 13 vs. 15 ± 15 g/d; *P* = 0.13). Thereafter, ALF had greater NDF intake than LFP, from wk 8 (64.6 ± 12.6 vs. 34.9 ± 14.9 g/d; *P* < 0.01) until wk 12 (246 ± 15 vs. 128 ± 17 g/d; *P* < 0.01). Forage NDF followed a similar trajectory, where ALF and LFP forage NDF intakes were similar until wk 8. Forage NDF in ALF was higher than LFP calves beginning in wk 9 (86 ± 9 vs. 24 ± 10 g/d; *P* < 0.01) until wk 12 (141 ± 11 vs. 30 ± 13 g/d; *P <* 0.01).

Similarly, average daily gain was not different between treatments (*P* = 0.61). Age at weaning readiness tended to be higher for LFP compared to ALF (*P* = 0.08; Table 1). There was no difference in feed efficiency between ALF and LFP groups (0.51 ± 0.05 vs. 0.47 ± 0.03 kg_gain_:kg_intake_, *P* = 0.24).

### 3.2 Blood and rumen parameters

**Fig. 3:**
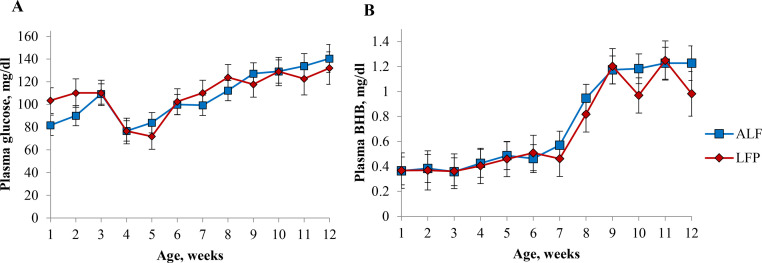
Plasma glucose concentration (A) and βHB concentration (B) of pre-weaned Jersey calves provided with long-stem alfalfa hay either ad libitum (ALF) or limited forage provision at 90 g/d, as fed (LFP). All calves were individually housed in hutches inside a pole barn, using sand as bedding. All calves were fed 1200 g/d of milk replacer and had ad libitum access to water and calf starter. Plasma glucose and BHB were not significantly different between treatments (*P* = 0.43 and *P* = 0.38, respectively).

Plasma glucose (*P* = 0.43) and BHB (*P* = 0.69) concentrations were not different across treatments (Fig. 3). During the outbreak of salmonellosis after wk 3, plasma glucose in ALF and LFP dropped from 109.3 ± 8.9 and 110.24 ± 11.1 mg/dL, respectively, to 84.0 ± 8.9 and 71.9 ± 11.3 mg/dL in wk 5 before recovering to 112.3 ± 8.9 and 110.2 ± 11.3 mg/dL in wk 8. As starter intake increased starting in wk 8, BHB concentrations increased from a low at wk 7 of 0.57 ± 0.11 and 0.46 ± 0.14 mg/dL for ALF and LFP to a high of 1.23 ± 0.13 and 1.25 ± 0.16 mg/dL in wk 11.

At weaning readiness, when rumen pH was measured continuously, proportional hay intake was lower in LFP than in ALF (*P =* 0.01; Table 1). Forage NDF intake was lower in LFP than in ALF (*P* < 0.01), as was total NDF intake (*P* = 0.01). Total starch intake was not different between ALF and LFP (*P* = 0.37).

Total SCFA concentration did not differ between ALF and LFP treatments (Table 2). Except for butyrate, proportions of individual SCFA were not different between ALF and LFP. Butyrate proportion tended to be greater in ALP calves compared to LFP calves (*P* = 0.06). Mean rumen pH tended to be greater in ALF than LFP calves (*P* = 0.09; Table 2), while LFP calves experienced longer duration of sub-acute ruminal acidosis (*P* = 0.03) and a higher area under the curve (*P* = 0.02).

**Table 2:** Rumen SCFA and pH profile^1^ of pre-weaned Jersey calves consuming at least 680 g/d of calf starter for three consecutive days. Calves^2^ were provided with long-stem alfalfa hay either ad libitum (ALF) or limited forage provision at 90 g/d, as fed (LFP).

	ALF^3^	LFP^3^	*P*-Value
Total, m*M*	87.7 ± 13.1	62.1 ± 16.7	0.16
Acetate,% total	64.0 ± 3.7	63.5 ± 4.9	0.90
Propionate,% total	16.7 ± 2.7	21.6 ± 2.7	0.19
Butyrate,% total	12.3 ± 2.7	5.8 ± 3.6	0.06
A:P Ratio	4.0 ± 0.6	3.3 ± 0.7	0.36
			
Isobutyrate,% total	1.5 ± 0.3	2.0 ± 0.4	0.37
Isovalerate,% total	2.6 ± 0.6	3.8 ± 0.6	0.11
Valerate,% total	1.8 ± 0.2	2.0 ± 0.3	0.43
Isocaproate,% total	0.1 ± 0.01	0.1 ± 0.01	0.84
Caproate,% total	0.7 ± 0.2	0.7 ± 0.2	0.89
			
Minimum pH	4.88 ± 0.35	4.18 ± 0.53	0.17
Mean pH	6.38 ± 0.16	5.98 ± 0.23	0.09
Maximum pH	7.25 ± 0.13	7.11 ± 0.20	0.40
Standard deviation	0.26 ± 0.02	0.29 ± 0.03	0.45
Duration pH < 5.8, min/d	261 ± 133	796 ± 145	0.03
Area under curve pH < 5.8, pH × min/d	60 ± 43	249 ± 47	0.02

1 Rumen pH measured every 2 min for 7 days. Daily profiles were created, and all 7 daily profiles were averaged per calf.

2 All calves were individually housed in hutches inside a pole barn, using sand as bedding. All calves were fed 1200 g/d of milk replacer and had ad libitum access to water and calf starter.

3 n = 6.

### 3.3 Transporters and regulators of intracellular pH

Between the ALF and LFP treatments, no difference was found in MCT1 abundance (*P* = 0.24; Table 3), NBC1 abundance (*P* = 0.77), or NHE3 abundance (*P* = 0.13).

**Table 3:** Transporter abundance of post-weaned Jersey calves. Calves^1^ were provided with long-stem alfalfa hay either ad libitum (ALF) or limited forage provision at 90 g/d, as-fed (LFP).

	ALF	LFP	*P-*Value
MCT1, AU	1272 ± 239	858 ± 239	0.24
NBC1, AU	928 ± 137	783 ± 162	0.77
NHE3, AU	1003 ± 145	614 ± 171	0.13

1 All calves were individually housed in hutches inside a pole barn, using sand as bedding. All calves were fed 1200 g/d of milk replacer and had ad libitum access to water and calf starter.

## 4 Discussion

### 4.1 Impact of solid feed intake on rumen pH

Rumen pH in the calf is lower than in the adult cow, but increases with age (Khan et al., 2007, Khan et al., 2008). The introduction of grain-based calf starter and chopped timothy hay or mixed legume hay increases ruminal SCFA concentrations by 50 % without the typically-seen drops in rumen pH (Laarman et al., 2012), or changes in SCFA transport from the rumen (Yohe et al., 2019). In our study, we saw consumption of grain-based calf starter increase over time, regardless of treatments, and likely resulting from the high milk allowance in this study. Simultaneously, DMI as a percent of body weight was significantly lower for LFP than for ALF, suggesting lower long-stem alfalfa hay intake was not compensated for by higher calf starter intake. Altogether, this indicates that growth performance in the developing calf is resilient to changes in long-stem alfalfa hay provision.

Since starter intake was not different between LFP and ALF calves, differences in rumen pH were likely due to differences in provision of long-stem alfalfa hay. The principal factor driving calf starter intake is milk allowance, which explains why the weaning readiness was not reached until 82 – 96 days of age. Regardless of milk allowance, feeding chopped hay increases rumen pH in both pre- and post-weaned Holstein calves (Laarman & Oba, 2011; Laarman et al., 2012). Because dry forage intake is likely relative to grain-based calf starter consumption (Suarez-Mena et al., 2016), and the LFP calves did not reach their long stem alfalfa hay limit until wk 10, it is likely differences in rumen pH due to dry forage consumption are not exhibited until ad libitum dry forage intake exceeds 90 g/d as-fed. Suarez-Mena et al. (2016) suggested that forage provision may be beneficial to calves when they are experiencing SARA, and provide no benefit when calves are not experiencing SARA. Despite the occurrence of SARA in this study, the adverse effects of SARA in adult cows were absent in calves, in line with another recent study (McCurdy et al., 2019).

### 4.2 Impact of rumen pH on SCFA transport

A decrease in rumen pH may even be beneficial to the pre-weaned calf by driving the necessary remodeling to improve absorption capacity (Hiltz et al., 2021). In adult cows, the transition to a higher concentrate ration post-partum involves the acidification of ruminal epithelial cells as part of epithelial remodeling (Laarman et al., 2015). Our results are inconsistent with those in cows fed supplemented butyrate, where increased MCT1, NHE3, and NBC1 expression over time suggest a homeostatic mechanism to restore the intracellular pH balance (Laarman et al., 2013). Our results are more consistent with a recent weaning study in calves, where weaning, but not calf starter intake or lower rumen pH, decreased expression of NBC1, an intracellular pH regulator in epithelial cells, suggesting an intracellular acidification (Hiltz et al., 2021). In our study, however, there was no change in the transporter abundance between ALP and LFP treatments, suggesting the acidification adaptive mechanism is not occurring pre-weaning under this feeding regimen, despite the more acidic rumen environment. The results adding to the indication that calves either lack the ability to adapt to rumen pH changes or have no need to adapt; however, due to the limited statistical power, it is possible that smaller differences in transporter expression were present but undetected.

### 4.3 Limitations

One limitation of our study was that our measures were collected by starter intake rather than calf age, resulting in variability in age at harvest. Limit-fed calves tended to have an older weaning age, suggesting that forage provision could better prepare calves for weaning. Our data complements other studies found that forage provided to calves promotes rumen development by increasing rumen capacity and volume without compromising nutrient digestibility or utilization (Castells et al., 2012; Khan et al., 2011). This indicates that both forage and starter are needed to develop the best diet to meet the needs of the growing and developing calf, and that both can accelerate the weaning process.

Another limitation of our study is the low animal number post-*Salmonella* outbreak. While the study started with 21 animals (ALP *n* = 12; LFP *n* = 9), incidence of mortality, including a detection of salmonellosis in four of nine deceased calves and six of twelve surviving calves left our study with 12 animals total (ALP *n* = 6; LFP *n* = 6). With the remaining power, we were able to detect 20 % differences between treatments with 80 % power. Therefore, our results would not detect smaller mean differences, thus should only focus on the major differences found in these data.

## 5 Conclusion

In pre-weaned Jersey calves, limited provision of long-stem alfalfa hay was sufficient to maintain starter intake, ADG, plasma glucose and BHB, SCFA concentrations, and transporter abundance, as indicators of calf growth and rumen development. Limiting forage provision showed a decline in rumen pH, indicating that an increased forage provision helps maintain a more neutral rumen pH in pre-weaned Jersey calves. However, the decrease in rumen pH did not impair the markers measured for rumen development both in this study and in others or impact growth rates. Thus, re-evaluating our definition of SARA and our understanding of how depressed rumen pH impacts, or may not impact, performance in pre-weaned calves is required. As the definition stands, subacute ruminal acidosis in calves does not cause the same decline in performance seen in lactating cows.

## Funding

Anne H. Laarman reports equipment, drugs, or supplies was provided by Calva Products. Anne H. Laarman reports financial support was provided by USDA. Anne H. Laarman reports financial support and administrative support were provided by Idaho Agricultural Experiment Station. Anne H. Laarman reports financial support was provided by American Jersey Cattle Association and National All-Jersey Inc. If there are other authors, they declare that they have no known competing financial interests or personal relationships that could have appeared to influence the work reported in this paper.

## CRediT authorship contribution statement

**Madeline N. Bennett:** Writing – review & editing, Writing – original draft, Formal analysis. **Dana E. McCurdy:** Writing – original draft, Methodology, Investigation, Formal analysis. **Anne H. Laarman:** Writing – review & editing, Supervision, Funding acquisition, Formal analysis, Conceptualization.

## Declaration of competing interest

The authors declare that they have no known competing interests.
